# Dielectric Constant Modelling with Soil–Air Composition and Its Effect on Sar Radar Signal Backscattered over Soil Surface

**DOI:** 10.3390/s8116810

**Published:** 2008-11-01

**Authors:** Mehrez Zribi, Aurélie Le Morvan, Nicolas Baghdadi

**Affiliations:** 1 CETP/IPSL/CNRS, 10-12 av. de l'Europe, 78140 Vélizy, France; 2 IRD-CESBIO, 18 av. Edouard Belin, bpi 2801, 31401 Toulouse cedex 9, France; 3 CEMAGREF, UMR TETIS, 500 rue François Breton, 34093 Montpellier cedex 5, France

**Keywords:** dielectric constant, radar, moisture, modelling

## Abstract

The objective of this paper is to present the contribution of a new dielectric constant characterisation for the modelling of radar backscattering behaviour. Our analysis is based on a large number of radar measurements acquired during different experimental campaigns (Orgeval'94, Pays de Caux'98, 99). We propose a dielectric constant model, based on the combination of contributions from both soil and air fractions. This modelling clearly reveals the joint influence of the air and soil phases, in backscattering measurements over rough surfaces with large clods. A relationship is established between the soil fraction and soil roughness, using the Integral Equation Model (IEM), fitted to real radar data. Finally, the influence of the air fraction on the linear relationship between moisture and the backscattered radar signal is discussed.

## I. Introduction

Soil moisture and roughness parameters play a key role in hydrological and climate studies. Considerable effort has been devoted to the study of radar backscattering responses from natural surfaces, in active microwave remote sensing [1-5]. Firstly, electromagnetic backscattering models (Kirchoff models, the small perturbation Model) [1] and more recently, the Integral Equation Model (IEM, [6]) have been used in order to estimate these parameters. However, different experimental measurements have shown that their use should be restricted to smooth or medium-rough surfaces. For example, the Small Perturbation Model (SPM) is valid only for very smooth soils, IEM shows a validity domain with k s<1.3 (k: wave number, s: rms height [7]). In practice, it is still difficult to identify a single model with a large domain of validity, for real agricultural soils, in spite of the improvements achieved over the past few years [8, 9]. An approach, based in the parameterisation of a correlation length function of the rms height, has been proposed in order to improve the fit of IEM to real radar data in different configurations [4, 5, 10]. Various improvements have been achieved in the description of roughness, by introducing multi-scale approaches [11, 12], generalized power law spectra [13], and by introducing new parameters such as the Zs index [3]. However, even with these improvements, discrepancies are still observed for medium or high degrees of roughness [14].

Secondly, past experimental studies have been designed to estimate surface moisture only, by adding different hypotheses to the surface roughness description. A linear relationship between surface moisture and backscattered radar signals has been proposed [1]. This approach was validated by a large number of experimental studies [15, 16]. It is generally considered to be a valid approximation for a given studied site. However, the coefficients needed to describe the linear relationship often vary from one watershed to another and also from one year to another, and thus need to be calibrated [16, 17]. For this reason, it is still very difficult to use this relationship for radar signal inversion, without making a considerable effort to obtain correct calibration of the coefficients.

In general, for agricultural soils with rough surfaces, we observe clods with a large air fraction between them. This air fraction is not taken into account when estimating the dielectric constant. In fact, with the gravimetric approach, and thetaprobe or TDR measurements, we neglect this effect and analyse the soil fraction only. In the present study, we propose a new modelling process for the dielectric constant, in order to include the effects of the air fraction. Our calculation of the dielectric constant is based on the assumption of a composite ground, with both soil and air fractions. Section 2 describes the experimental data used for this work, recorded during three experimental campaigns (Orgeval'94, Pays de Caux'98, Pays de Caux'99). Section 3 describes IEM modelling, using dielectric constant modelling based on the introduction of both “air” and “soil” phases. It presents an analysis of the relationship between soil fraction and radar signal behaviour, and its influence on the relationship between radar signal and soil moisture. Finally, our conclusions are presented in Section 4.

## II. Experimental campaigns

In this study, data was acquired during three experimental campaigns (Orgeval'94, Pay de Caux'98, 99,), over agricultural watersheds. For each one of these, SAR images (RADARSAT-1, SIR-C) were acquired with different configurations (Table 1). Simultaneously to the radar acquisitions, ground measurements were carried out in a large number of test fields. Measurements of the soil moisture, within the top 5 cm, were made using a gravimetric method and/or a TDR probe. Roughness measurements were carried out using a pin-profiler (with a total length of 1 and 2 m, and a resolution of 5 and 10 mm, respectively). In order to ensure that sufficient statistical precision could be achieved, ten profiles were taken for each test field.

### 1- Orgeval'94

The objective of this experimental campaign was to characterize the effect of soil roughness on radar measurements. The Orgeval watershed is located to the east of Paris (France; Lat. 48° 51′N and Long. 3° 07′E). The experimental campaign was conducted by the CETP (Centre d'étude des Environnements Terrestre et Planétaires) and the CEMAGREF (Centre d'Etude de Machinisme Agricole, du Génie Rural et des Eaux et Forets). The Orgeval'94 campaign was run concurrently with the SIRC/XSAR (1.25 GHz, 5.3GHz and 9.25 GHz) missions [6].

The soil composition is relatively constant over the whole basin: 17% clay, 78% silt, and 5% sand. Simultaneous ground measurements were carried out in eight bare soil fields. The soil moisture content remained high and constant over the watershed (about 0.35 cm^3^/cm^3^), such that the soil roughness and tilling practices were the only remaining factors, which could influence the backscattered radar signals.

### 2- Pays de Caux'98, 99

The Pays de Caux'98, 99 experimental campaign was designed to characterize the agricultural soil roughness and its effects on erosion and runoff in the North of France. The test site was the Blosseville watershed located in the Pays de Caux, in Northern France (long. 0°50′ W, lat. 49°47′ N). The loamy soils of the northern European loess belt are sensitive mainly to soil structure degradation, and are commonly exposed to erosion caused by concentrated runoff. Within that framework, an intensive research program was carried out in the region of the Pays de Caux. The site's soil features are: (1) a very homogenous loamy texture (13% clay, 65% loam, and 22.5% sand), (2) approximately 50% of the fields are bare, or nearly bare. RADARSAT-1 measurements were recorded in 1998 and 1999, over more than 10 large (more than 100m x 100m each) test fields.

### 3- Roughness measurement results

**Figure 1.**
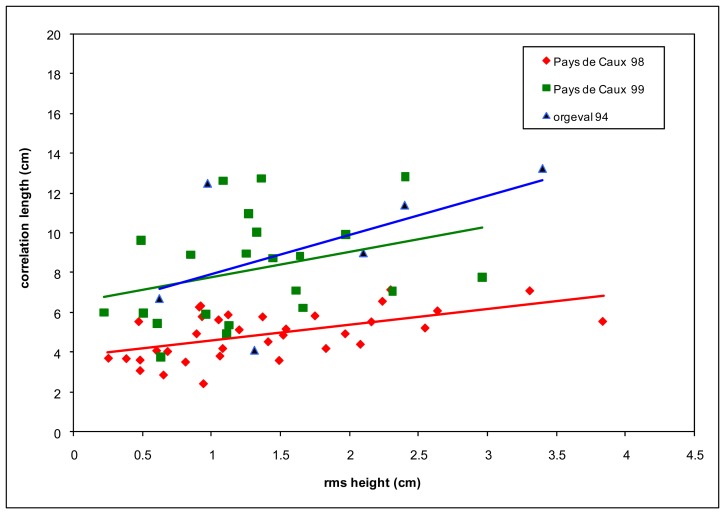
Roughness parameters of the test fields.

The rms heights (*s*) and correlation lengths (*l*) are extracted from the correlation function, computed from the measured soil profiles. Roughness measurements were made using a pin profiler (with a total length of 2 m and a resolution of 1 cm). The results illustrated in figure 1 show many types of agricultural soil, with *s* values ranging from 0.3 to 3.8 cm, and *l* ranging from 2.1 cm to 13.8 cm. We observe a correlation between these two parameters, for the studied test fields, with a tendency for the rms height to increase with correlation length, as illustrated by regression lines for the three databases.

## III. Backscattering modelling

### 1- IEM

The present study is focussed on a particular radar configuration: HH polarization and C band (5.3 GHz) operating mode, for the radar acquisitions. IEM is used as a reference, for the simulation of backscattering coefficients. It is the most used model in backscatter studies of bare soil ([18-19]). The IEM input parameters are the dielectric constant (deduced from the surface volumetric moisture content and soil texture), the surface height and correlation function or corresponding spectrum. Many studies have been made in order to derive a good and current description of the correlation function shape ([13-14], [20]). However, in the case of agricultural soil studies, an exponential correlation function generally provides a good fit to the majority of experimental surfaces ([21-23]). An exponential correlation function was therefore used in this study. In order to remain strictly within the domain of validity of the IEM and to prevent it from being affected by other influences resulting from volumetric scattering, we took into account only those surfaces with **k s** < 1.3 (k: wave number, s: rms height).

The dielectric constant is introduced into IEM, through the Fresnel reflection coefficients, *R*_0_, between the air (region 0) and the soil layer (with permittivity *ε*), which are defined as:

(1) $$R_{0}^{h}=\frac{\cos\theta -\sqrt{{ɛ}_{1}-\sin^{2}\theta }}{\cos\theta +\sqrt{{ɛ}_{1}-\sin^{2}\theta }}$$

(2) $$R_{0}^{v}=\frac{{ɛ}_{1}\cos\theta -\sqrt{{ɛ}_{1}-\sin^{2}\theta }}{{ɛ}_{1}\cos\theta +\sqrt{{ɛ}_{1}-\sin^{2}\theta }}$$

Water content is converted to a dielectric constant, by using a dielectric mixing model ([24]). These models need soil moisture and composition. The subscripts *h* and *v* denote horizontal or vertical polarisation, respectively. This IEM version introduces the transitional function proposed by Wu et al. [9], in which the Fresnel coefficient is not evaluated at the incidence angle *θ_i_*, but at an angle *θ* ranging between *θ_i_* and normal incidence, depending on the surface roughness parameters.

**Figure 2.**
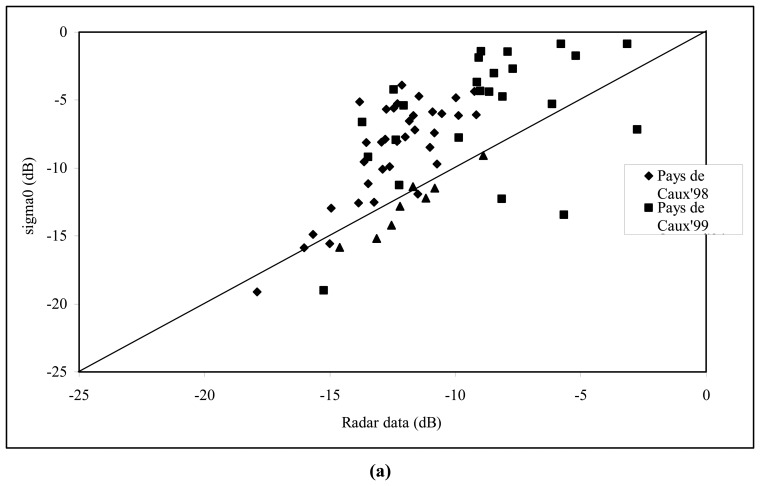

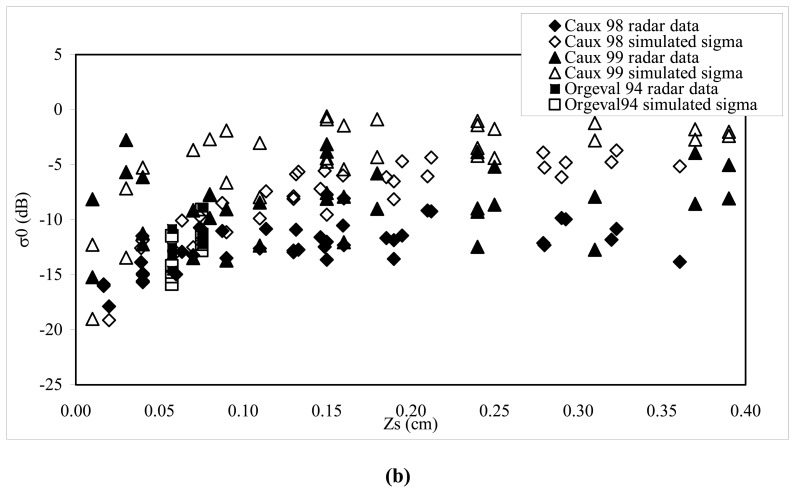
Inter-comparison between radar signal measurements and backscattering simulations as a function of soil roughness.

Figure 2-a provides a comparison between simulations and radar measurements, acquired over different test fields, showing good agreement for low values of radar signals in the drawn ellipse, corresponding to small roughness values. Radar signals with simulated values are illustrated in Figure 2-b as a function of the roughness parameter Zs.

Zribi and Deschambre [3] proposed this parameter, defined by *s*^2^/*l*, which is given by the product of the rms height (*s*) and the slope of the soil surface (*s*/*l*). Smooth soils correspond in general to small values of Zs (<0.1 cm), whereas ploughed soils correspond to large values of Zs (>0.1 cm). The discrepancy, between simulated and measured radar signals, increases when the values of the roughness parameter, Zs, reach higher values. This section shows particularly the difficulty of IEM to simulate correctly backscattering over surfaces with highest roughness, even in its validity domain. In the next sections, we present a hypothesis, concerning dielectric constant modelling, which could explain this disagreement for high roughness.

### 2- Dielectric constant modelling

**Figure 3.**
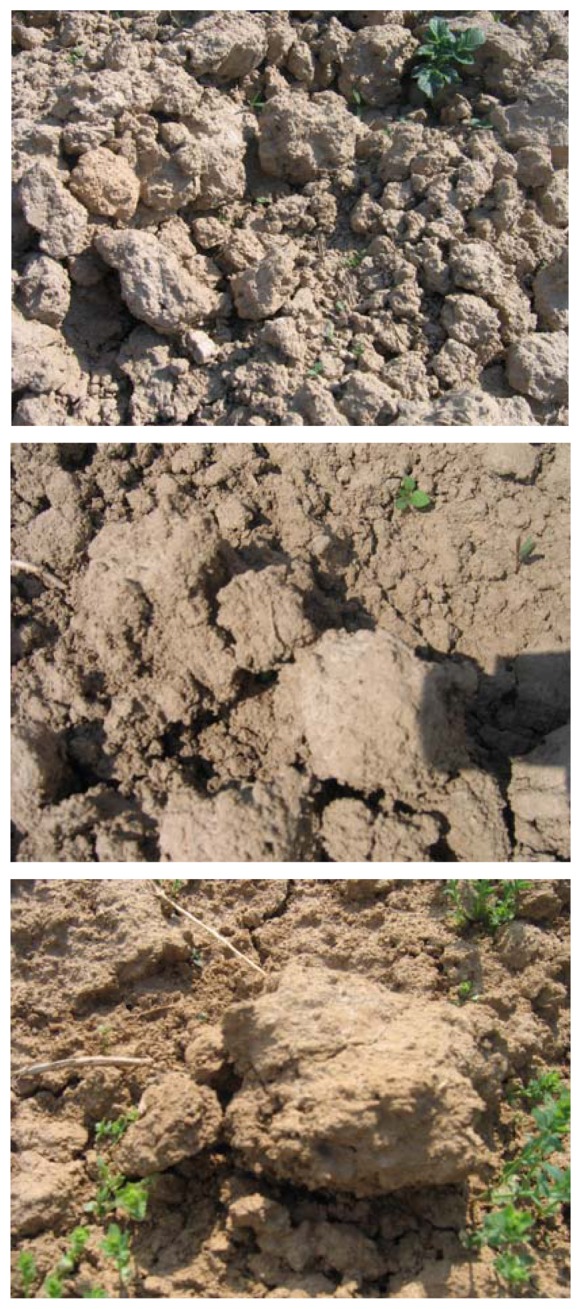
Illustration of real agricultural soils.

**Figure 4.**
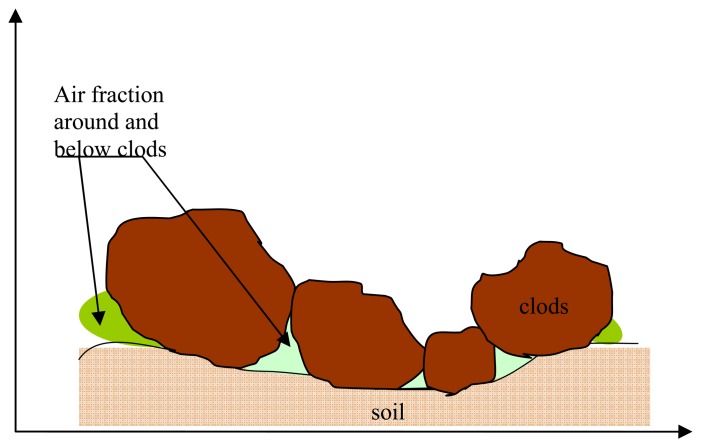
Clod illustration, showing soil and air fractions.

As can be seen in Figure 3, the agricultural soil surface is composed of clods of different sizes, embedded together, with considerable volumes of air between the clods. The presence of air is also observed around and below the clods (Figure 4). When determining the soil's dielectric constant, this air is not taken into account by the dielectric mixing model, which is more specifically adapted to homogenous soils with a smooth surface. In fact, as a general rule, measurements are made without including the presence of air between the clods. In the present case the surface backscattering models, which assume a homogenous soil texture, are not realistic for a large number of surfaces with medium or high roughness.

In this study, rather than trying to simulate the heterogeneity resulting from the presence of air, with an accurate 3D backscattering model, we introduce a modification to the modelling of the dielectric constant. We consider a dielectric, two-phase mixing model [25] for the “air” and “soil” phases. This type of decomposition is proposed by [26] for passive microwave measurement analysis. These phases are accounted for by their volume fractions (1-*v*_soil_) and *v*_soil_ in the mixing formula, yielding:

(3) $${ɛ}_{\text{app}}={\left[{v_{\text{soil}}}^{*}{{ɛ}_{\text{soil}}}^{\alpha }+{\left(1-v_{\text{soil}}\right)}^{*}{{ɛ}_{\text{air}}}^{\alpha }\right]}^{1/\alpha }$$

where the exponent α is set to α=0.5, representing the refractive mixing model ([27]).

In order to retrieve the variations of *v*_soil_ for our database, it was decided to fit IEM to the radar data, in the HH and C band configurations.

**Figure 5.**
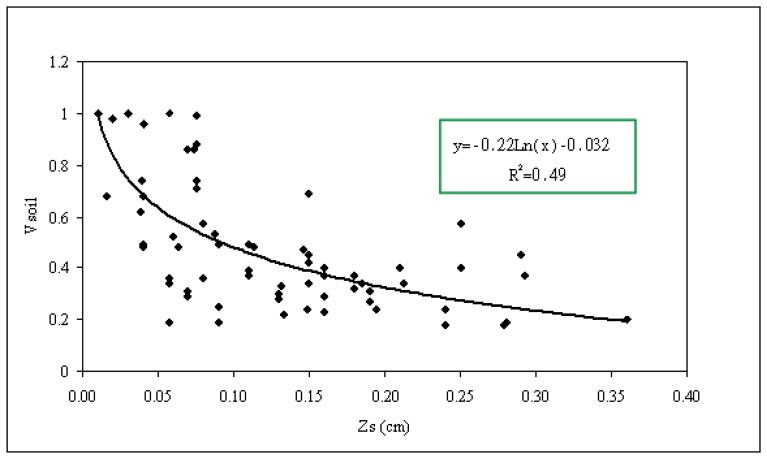
Variation of the *v*_soil_ parameter (soil fraction) as a function of the soil roughness parameter Zs.

Figure 5 shows how the *v*_soil_ parameter varies as a function of the roughness parameter Zs. A correlation can be observed between the soil fraction and surface roughness (correlation coefficient R^2^ equal to 0.49). This could be explained simply by the fact that, with increasing roughness, we have an increase in the number and size of clods and thus a higher probability of encountering a higher air fraction. We propose the following empirical relationship between Zs and *v*_soil_, derived from the fitted data:

(4) $$v_{\text{soil}}=-0.22\log(Zs)-0.032$$

Real data fitted to this model shows a very high portion of air (even superior to 0.7) for high roughness values. This is due, firstly to our use of a single dielectric constant and the hypothesis of homogeneous soil texture, and secondly, to the use of a surface model, which is not necessarily well adapted to the backscattering estimation, over these surfaces.

**Figure 6.**
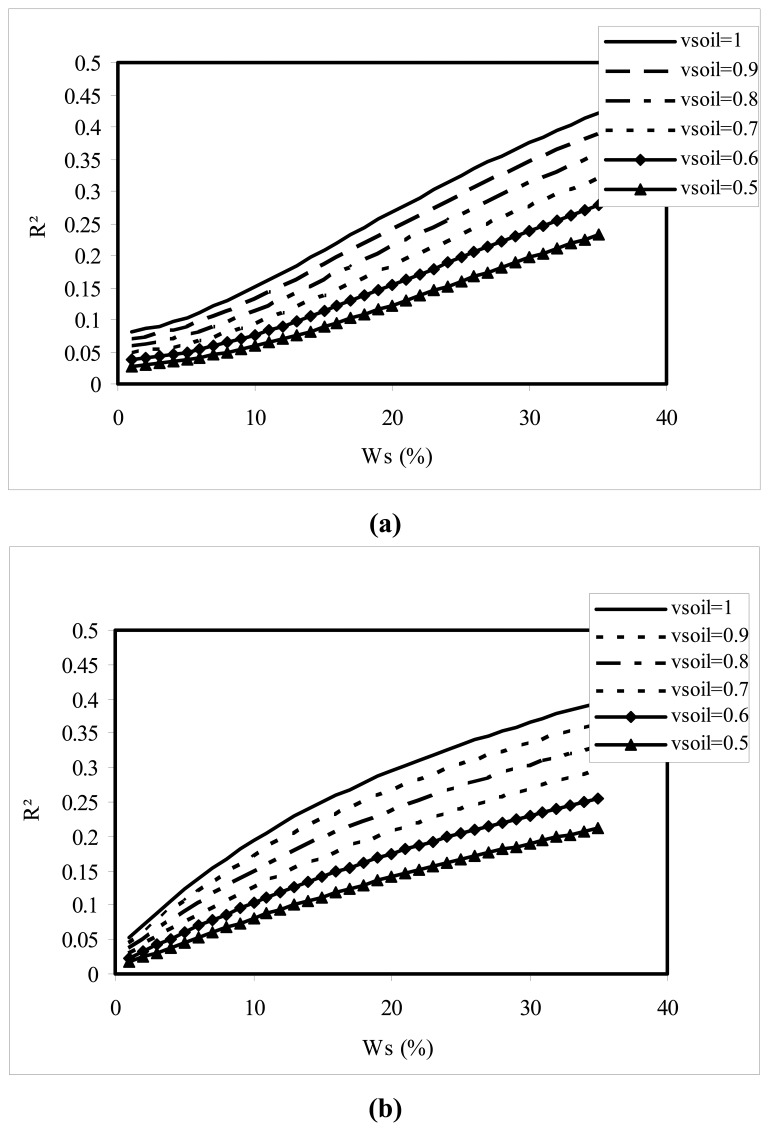
Illustration of variations in |*R*^2^|, as a function of soil moisture, for different values of the *v*_soil_ parameter (*v*_soil_=1, *v*_soil_ =0.9, *v*_soil_ =0.8, *v*_soil_ =0.7, *v*_soil_ =0.6), **(a)** at L band (1.25 GHz), **(b)** at C band (5.3 GHz).

Figure 6 illustrates the variation of the Fresnel coefficient square |*R*^2^|, on a linear scale, in HH polarization, in the L (1.25 GHz) and C (5.3GHz) bands, as a function of the volumetric moisture parameter *W_s_*, for different values of *v*_soil_. We clearly observe the strong influence of this parameter on the Fresnel coefficient, with increasing values of |*R*^2^| for a low air fraction. This could explain, in particular, the strong variations in radar signal observed as a result of variations in moisture, from one field to another. In fact, the saturation of |*R*^2^| (on a logarithmic scale) and of the resulting backscattered signal, at high moistures, will occur more rapidly when the air fraction is low. This is the reason for which backscattering simulations generally predict saturation, before it occurs in real measurements [15].

### 3- Analysis of the correlation between backscattering and moisture measurements

**Figure 7.**
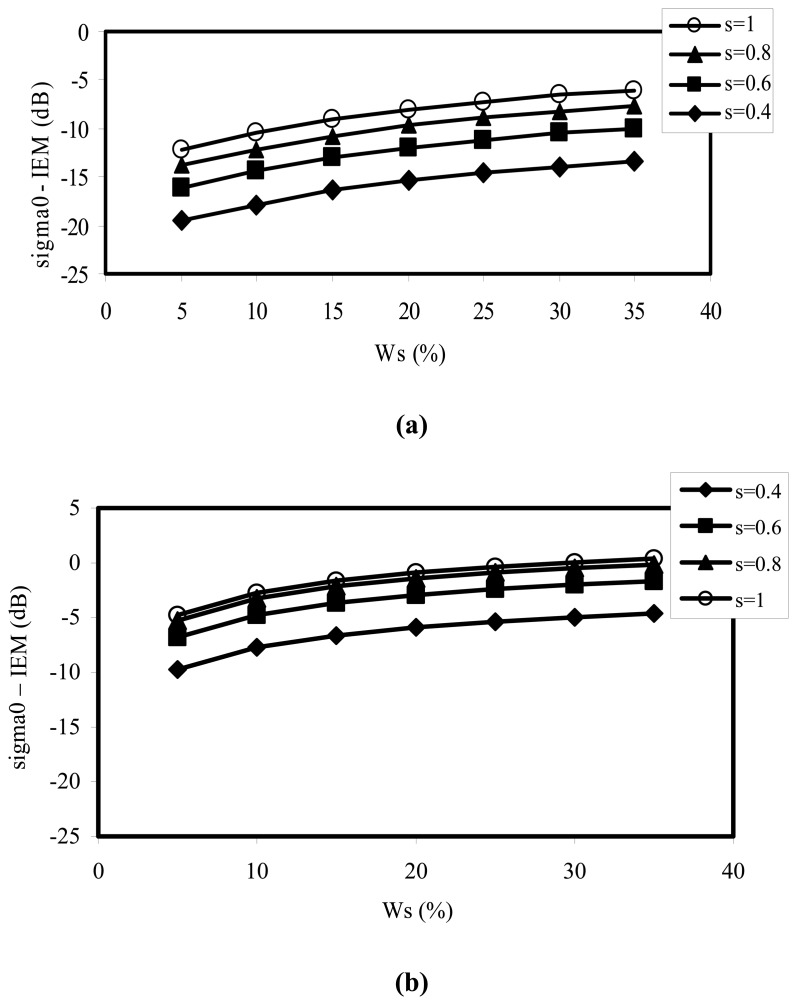
Illustration of IEM simulations, with a classical computation of the dielectric constant, for different values of roughness, as a function of soil moisture. **(a)** L band (1.25 GHz), **(b)** C band (5.3 GHz).

Figure 7 illustrates the variation of backscattering as a function of soil moisture, using IEM simulations, with no modification to the dielectric constant calculation, for the L and C bands. We observe, in particular, a saturation of the simulated signals for volumetric moisture of around 25%. This is not the case for real data, which generally exhibits a linear trend for volumetric moistures up to approximately 35%. On the other hand, in the past, the inversion of radar signals was generally based on a simple linear relationship between the radar signal at a given incidence angle (σ^0^), and the mean soil surface volumetric moisture (***W****_s_*):

(5) $$\sigma^{0}(dB)=a.W_{s}(\%)+b$$

where the parameters *a* and *b* depend on incidence angle, roughness and polarisation.

**Figure 8.**
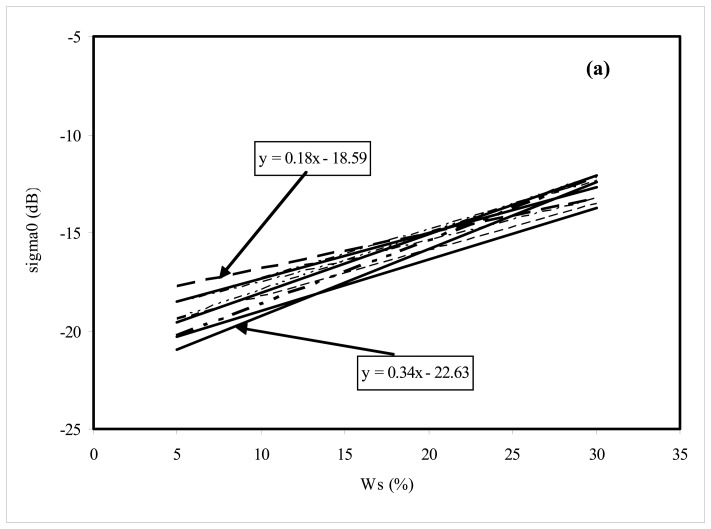

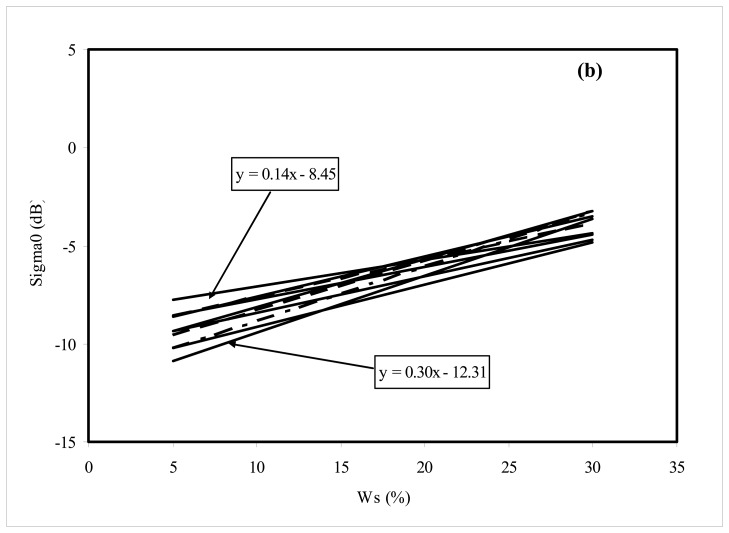
Illustration of the effects of air fraction on the linear relationship between soil moisture and simulated radar signals, lines corresponding to ten linear relationship cases, with for each one, as input, successive surface moisture values (5%, 10%,15%, 20%, 25% and 30%) with six *v*_soil_ parameters (*v*_1_, *v*_2_, *v*_3_, *v*_4_, *v*_5_, and *v*_6_) estimated from a uniform distribution between the values 0.6 and 1. **(a)** L band (1.25 GHz), **(b)** C band (5.3 GHz).

As described in the Introduction, the coefficients describing the linear relationship between moisture and radar signal are often different from one watershed to another and also from one year to another. For example, with ERS/SAR (VV polarisation, 23° incidence angle), the slope is equal to 0.33 for [16], equal to 0.28 for [15], equal to 0.42 for [28], equal to 0.55 for [29]. In this section, we propose to analyse the effect of air fraction percent on soil composition on the variation of the linear relationship regression coefficients (*a* and *b*). In order to understand this influence of the air fraction on *a*, *b*, we analysed various different case studies based on IEM simulations. We monitored the surface moisture of one site, on six different dates, corresponding to the successive surface moisture values: *W_s_*_1_=5%, *W_s2_*=10%, *W_s3_*=15%, *W_s4_*=20%, W*_s5_*=25% and W*_s6_*=30%. On the other hand, we assumed six *v*_soil_ parameters: *v_1_, v*_2_, *v*_3_, *v*_4_, *v*_5_, and *v*_6_, estimated from a uniform distribution between the values 0.6 and 1. These lead to infinite cases, which could be observed at real agricultural sites. We then simulated backscattering coefficients with IEM for different situations, by mixing one *v*_soil_ parameter (between 0.6 and 1) with one moisture value (*v*_l_, *W_sj_*, *i*=1,..6, *j*=1,..6), for six different dates. Linear relationships between the six input moistures and the corresponding backscattering coefficient IEM simulations were illustrated just for the ten cases in the L and C bands in Figure 8. The values of the coefficients *a* and *b* were found to be highly variable, with *a* in the L band ranging between 0.18 and 0.34 as illustrated in Figure 8-a, with a mean value equal to 0.26, and *a* in the C band ranging between 0.14 and 0.30 as shown in Figure 8-b, with a mean value of 0.22.

The influence of the air fraction can thus be clearly observed in the behaviour of the backscattered radar signals. This effect is more significant for low moistures, with a variation equal to 3 dB for a moisture value of 5%, and 2dB for a moisture value of 30%, in the C band (Figure 8-b).

This result explains the high variation of linear relationship between soil moisture and radar signal, due just to the presence of the air fraction in the estimation of dielectric constant. This is in agreement with the other experimental studies showing this variation [16, 17]. [30] have shown the effect of roughness in the variation of this relationship. The air fraction presence is certainly a second factor that could influence this relationship from one site to another, with differences due to the oldness of tillage, rain events, etc.

## IV. Conclusions

In this paper we deal with a scientific question, often neglected in our previous studies, related to the computation of the dielectric constant used in backscattering models for the estimation of soil roughness or moisture. For real agricultural soils, particularly for medium or rough surfaces, we have observed the presence of a significant air fraction that could be present between clods, or below clods. This portion could have a significant influence on the dielectric constant. In this study, based on a large experimental campaign involving moisture, roughness and radar measurements, we propose modelling of the dielectric constant in which both a soil fraction and an air fraction are used. The fitting of an IEM to real radar data clearly shows that an increase in roughness leads to an increase in the air fraction. However, this fraction could also be correlated to the time since the soil was last worked. Generally, for new soil tillage, we observe the presence of larger quantities of air between embedded clods, and with rainfall and runoff, the percentage of air decreases. Therefore, the *v*_soil_ parameter probably decreases with the age of the tillage. This effect could explain some of the differences in behaviour observed in the correlation between radar signals and soil moisture. These results illustrate the difficulty to analyse backscattering behaviour over natural surfaces. In future studies, first, it will be very interesting to estimate experimentally the percent of air fraction for different types of soil (large ploughed, ploughed, cloddy, smooth soils, etc) in order to propose a correction of dielectric constant computation, for each case of surface. Second, the consideration of volume diffusion in surface backscattering models needs to be more discussed and estimated, particularly for surfaces with large air fraction percent.

## Figures and Tables

**Table 1.** Details of radar data acquisition.

**Campaign**	**Sensor**	**Date**	**Configuration**
Orgeval 94	SIR-C	12/04/94 - 18/04/94	HH 44°,52°,55 °,57°
Pays de Caux 98	RADARSAT	24/02/98 09/02/98 08/02/98	HH 39° HH 47°
Pays de Caux 99	RADARSAT	07/02/99 04/02/99	HH 23° HH 40°
